# Risk factors for syphilis in women: case-control study

**DOI:** 10.11606/S1518-8787.2017051007066

**Published:** 2017-08-03

**Authors:** Vilma Costa de Macêdo, Pedro Israel Cabral de Lira, Paulo Germano de Frias, Luciana Maria Delgado Romaguera, Silvana de Fátima Ferreira Caires, Ricardo Arraes de Alencar Ximenes

**Affiliations:** I Programa de Pós-Graduação em Saúde da Criança e Adolescente. Departamento de Enfermagem. Universidade Federal de Pernambuco. Recife, PE, Brasil; II Departamento de Nutrição. Universidade Federal de Pernambuco. Recife, PE, Brasil; III Grupo de Estudos de Avaliação em Saúde. Diretoria de Ensino e Pesquisa. Instituto de Medicina Integral Prof. Fernando Figueira. Recife, PE, Brasil; IV Programa de Pós-Graduação em Medicina Tropical. Unidade de Neonatologia do Hospital das Clínicas. Universidade Federal de Pernambuco. Recife, PE, Brasil; V Departamento de Parasitologia. Universidade de Pernambuco. Recife, PE, Brasil; VI Departamento de Medicina Tropical. Universidade Federal de Pernambuco. Recife, PE, Brasil

**Keywords:** Pregnant Women, Syphilis Serodiagnosis, Risk Factors, Socioeconomic Factors, Maternal-Child Health Services, Case-Control Studies

## Abstract

**OBJECTIVE:**

To determine the sociodemographic, behavioral, and health care factors related to the occurrence of syphilis in women treated at public maternity hospitals.

**METHODS:**

This is a case-control study (239 cases and 322 controls) with women admitted to seven maternity hospitals in the municipality of Recife, Brazil, from July 2013 to July 2014. Eligible women were recruited after the result of the VDRL (Venereal Disease Research Laboratory) under any titration. The selection of cases and controls was based on the result of the serology for syphilis using ELISA (enzyme-linked immunosorbent assay). The independent variables were grouped into: sociodemographic, behavioral, clinical and obstetric history, and health care in prenatal care and maternity hospital. Information was obtained by interview, during hospitalization, with the application of a questionnaire. Odds ratios and 95% confidence intervals were estimated using logistic regression to identify the predicting factors of the variable to be explained.

**RESULTS:**

The logistic regression analysis identified as determinant factors for gestational syphilis: education level of incomplete basic education or illiterate (OR = 2.02), lack of access to telephone (OR = 2.4), catholic religion (OR = 1.70 ), four or more pregnancies (OR = 2.2), three or more sexual partners in the last year (OR = 3.1), use of illicit drugs before the age of 18 (OR = 3.0), and use of illicit drugs by the current partner (OR = 1.7). Only one to three prenatal appointments (OR = 3.5) and a previous history of sexually transmitted infection (OR = 9.7) were also identified as determinant factors.

**CONCLUSIONS:**

Sociodemographic, behavioral, and health care factors are associated with the occurrence of syphilis in women and should be taken into account in the elaboration of universal strategies aimed at the prevention and control of syphilis, but with a focus on situations of greater vulnerability.

## RESUMO

**OBJETIVO:**

Determinar os fatores sociodemográficos, comportamentais e de assistência à saúde relacionados à ocorrência de sífilis em mulheres atendidas em maternidades públicas.

**MÉTODOS:**

Trata-se de um estudo caso-controle (239 casos e 322 controles) com mulheres admitidas em sete maternidades do município do Recife, no período de julho de 2013 a julho de 2014. As mulheres elegíveis foram recrutadas após o resultado do VDRL (*Venereal Disease Research Laboratory*) sob qualquer titulação. A seleção dos casos e controles considerou o resultado da sorologia por ELISA (*enzyme-linked immunosorbent assay*), variável dependente utilizada como diagnóstico para sífilis neste estudo. As variáveis independentes foram agrupadas em: sociodemográficas; comportamentais; e antecedentes clínicos e obstétricos; e assistência à saúde no pré-natal e na maternidade. As informações foram obtidas por meio de entrevista, durante o internamento, por aplicação de um questionário. Foi calculado o*dds ratio* (OR), intervalo de confiança de 95% e realizada análise de regressão logística para identificar os fatores preditores da variável a ser explicada.

**RESULTADOS:**

A análise de regressão logística identificou como fatores determinantes para a sífilis gestacional: nível de escolaridade fundamental incompleto ou analfabeta (OR = 2,02), ausência de acesso a telefone (OR = 2,4), religião católica (OR = 1,70), quatro ou mais gestações (OR = 2,2), três ou mais parceiros sexuais no último ano (OR = 3,1), uso de drogas ilícitas antes dos 18 anos (OR = 3,0) e uso de drogas ilícitas por parte do atual companheiro (OR = 1,7). Além desses, foram observadas a ocorrência de apenas uma a três consultas ao pré-natal (OR = 3,5) e história anterior de infecção sexualmente transmissível (OR = 9,7).

**CONCLUSÕES:**

Fatores sociodemográficos, comportamentais e de assistência à saúde estão associados à ocorrência de sífilis em mulheres e devem ser levados em consideração na elaboração de estratégias universais direcionadas à prevenção e controle da sífilis, porém com foco em situações de maior vulnerabilidade.

## INTRODUCTION

Gestational syphilis is a public health problem in the world. It is estimated that approximately two million pregnant women present active infection each year and less than 10% are diagnosed and treated^1,8^. Approximately 90% of the cases occur in developing countries; however, we can observe resurgence in developed nations^2^.

The disease causes multiple adverse outcomes in pregnancy, with an estimated 4.5 times higher risk when compared to pregnant women without the diagnosis^10^. As it causes vertical transmission, it can cause miscarriage, premature delivery, and fetal and neonatal death if not treated properly. Newborns of mothers with syphilis who are untreated or inadequately treated may be asymptomatic. This can lead to the absence of diagnosis and treatment, causing serious damage to their health, with psychological and social repercussions^6^.

The recommendations for the control of the disease reinforce interventions aimed at the prevention and timely diagnosis, paying attention to more exposed population groups^9^. Numerous conditions have been associated with the occurrence of syphilis during pregnancy, including sociodemographic, behavioral, and health care factors^13^.

Among the sociodemographic factors, low education level, low income, and marital status (common-law marriage or living together) are identified as risk situations and an expression that syphilis is related to, but not limited to, poverty. Equally important are the behaviors that make women vulnerable, that is being associated with a higher risk, such as lower age of first sexual intercourse and pregnancy, high number of sexual partners, non-adherence to safe sex practices, use of illicit and psychoactive drugs, among others^8,13,18^. Some of these conditions increase the risk when related to the insufficient access to health services.

Brazil is signatory of international plans for the elimination of syphilis. Recently, the World Health Organization (WHO) has defined, as goals for the certification of elimination of syphilis, the 95% of coverage of prenatal care and screening for syphilis in pregnant women, in order to reach the goal of congenital syphilis of 0.5 cases per 1,000 live births, which has been reached only by Cuba and Chile in the Latin America^12^.

In the Brazilian scenario, gestational syphilis presents a high magnitude and most cases continue to be diagnosed late, especially in the North and Northeast regions, where difficulties persist in the control of the disease^a^. The recognition of the complex and dynamic determination of sexually transmitted infections and the deepening of the knowledge on risk factors, in the relation to sociodemographic, behavioral, life condition, and organizational changes in the health system and services, can contribute to the updating of interventions aimed at them^9,12^.

The objective of this study was to determine the sociodemographic, behavioral, and health care factors related to the occurrence of syphilis in women treated at public maternity hospitals.

## METHODS

This is a case-control study, carried out from July 2013 to July 2014, in seven maternity hospitals (six public administered and one philanthropic), corresponding to the total number of units in operation and that worked for the Brazilian Unified Health System (SUS) in Recife, capital of the state of Pernambuco, located in the Brazilian Northeast.

Eligible women were recruited after VDRL (Venereal Disease Research Laboratory) under any titration, admitted to these services because of labor, postpartum, abortion, and any other clinical-surgical complication of the puerperal pregnancy cycle. Women admitted to intensive care units (ICU) and those who did not demonstrate clinical or cognitive conditions to respond to the interview were not recruited.

The cases and controls lived in the municipality of Recife. The classification between cases and controls was established after serological diagnosis by ELISA (enzyme-linked immunosorbent assay), a confirmatory treponemal test, regardless of the result of the VDRL (Figure). This was necessary because VDRL is not a treponemal test and several factors may affect its results.

**Figure:**
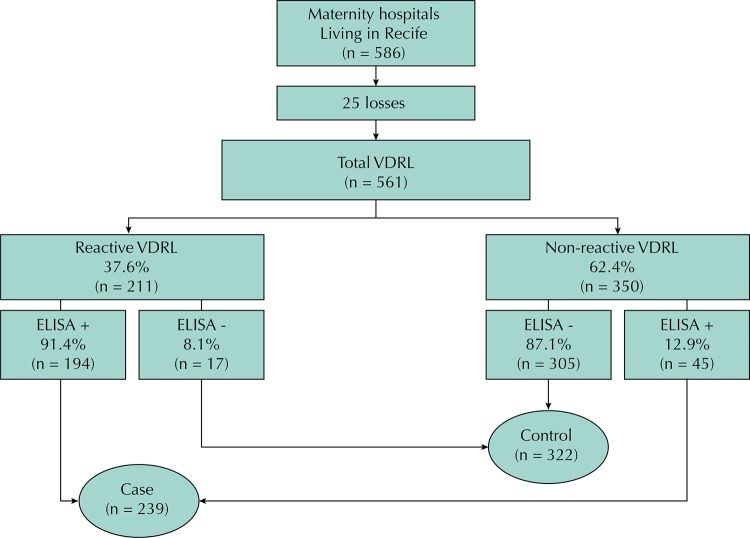
Flowchart of the participants in maternity hospitals. Recife, State of Pernambuco, Brazil, 2014.

VDRL: Venereal Disease Research Laboratory; ELISA: enzyme-linked immunosorbent assay

The sample size estimation was performed using the Stalcalc program of the EpiInfo statistical package, version 6.04, based on the study of Melo et al.^15^ We considered a 95% confidence level, power of 80%, and odds ratio (OR) of approximately 2.8 for the worst situation, according to socioeconomic, biological, and prenatal care indicators in the municipality of Recife (2004/2006). The ratio was one case to two controls and we considered the possibility of 10% of losses or refusals. Sample distribution considered the size of the health facility and the proportion of the number of beds available in obstetrics, according to data from the National Register of Health Establishments^b^ (CNES), varying from 6% (smaller) to 27% (bigger). Adopting these criteria, we estimated that we would need 480 women, being 160 cases and 320 controls.

Information was obtained by interview, during hospitalization, with a structured questionnaire applied by health professionals and university students from the area of health and social sciences. At the end, 2 ml of blood were collected for syphilis serology using the ELISA method, chosen because of its high sensitivity and specificity compared to other treponemal tests (TPHA, FTA-ABS) and because it does not suffer interferences in the analysis, as hemolyzed samples or with relative concentration of lipids can be processed^c^. The samples were initially stored in refrigerated maternity laboratories, identified with the research number, and transported short distances every two days to the Immunology and Parasitology Service of the *Instituto de Ciências Biológicas* of the *Universidade de Pernambuco* (UPE). To perform the ELISA, we used the Wiener Laboratories^®^ kit, which has recombinant *Treponema pallidum* antigens. Processing occurred according to manufacturer’s instructions.

The independent variables were grouped into: a) sociodemographic (age at the time of interview, marital status, race, household income *per capita* based on minimum wage (MW) at the date of the interview (R$724.00, equivalent to US$270), socioeconomic class (ABEP^d^), education level, paid work, insertion in the labor market, access to the Internet and telephone); b) behavioral (religion, age of first intercourse and first gestation, number of previous gestations and sexual partners, frequency of condom use, age at which started smoking, use of alcoholic beverages or illicit drugs, use of illicit drugs by current partner, feeling of discrimination (race, gender, financial situation), frequency of the use of television and radio, self-assessment on the access to information and situation of conflict (familiar, labor, or criminal); c) clinical, obstetric, and health care history in prenatal and maternity hospital (abortion history, previous sexually transmitted infection, number of prenatal appointments, gestational age and place where most appointments happened, availability of prenatal card from the first appointment and results of requests for laboratory tests, participation in group of pregnant women, registration in a family health unit, result of HIV serology in the maternity hospital).

The data were coded and double entered and EpiInfo’s VALIDATE program was used to check for inconsistencies (EpiInfo software, version 6.04). The variables of age at which started smoking, drinking, and using illicit drugs and their use in the gestational period presented high collinearity (Pearson: r > 0.8). We chose to include the variables of age at which started smoking, drinking, and using illicit drugs because of the greater explanatory power in the model and we excluded those that assessed their use in pregnancy.

In the program SPSS (Statistical Package for the Social Sciences), version 13, we performed the bivariate analyses between the potential risk factors and the outcome and estimated the odds ratios (OR) and respective 95% confidence intervals (95%CI). The variables that presented p-value ≤ 0.20 were included in the multivariate analysis model. At this stage, the hierarchical modeling process with levels was adopted as a strategy to introduce the variables, considering the possible risk factors for gestational syphilis. The last level was adjusted for the risk factors of levels 1 and 2. Thus, the variables of the last level did not influence the explanatory power of the previous levels.

For the unadjusted and adjusted OR and their respective 95%CI, we defined as the reference category the one with the lowest risk for gestational syphilis in the studied sample and we considered p ≤ 0.05 as significant.

The study was approved by the Ethics Committee of the Health Sciences Center of *Universidade Federal de Pernambuco* (Protocol 136.500, of Nov/2012). We took every precaution to ensure the confidentiality and confidentiality of the information. Before each interview, we obtained the written acceptance of the participants after reading the informed consent.

## RESULTS

Of the 586 potentially eligible women identified in the participating maternity hospitals, we had 25 losses because of serological inadequacies of the VDRL. Thus, 239 cases and 322 controls were included (Figure).

The participants presented similarity between age groups, with approximately 25% for each category considered. Regarding marital status, common-law marriage prevailed, with 62%; married women accounted for only 9%. Regarding education level, 37% had not finished basic education or were illiterate and 28% had completed high school. Most women (40%) reported having a household income per capita between 1/2 and 1/4 of the minimum wage, and only 9% had a higher pay. Most women did not use male or female condom in sexual intercourse.

Table 1 shows the distribution of the main sociodemographic variables for cases and controls, with their corresponding crude OR and 95%CI. The variables selected for multivariate analysis (p ≤ 0.20) were: marital status, self-reported race, education level, economy class, and access to the Internet and telephone.

**Table 1:** Bivariate analysis of sociodemographic factors for syphilis in women. Recife, State of Pernambuco, Brazil, 2014.

Variable	Total		Case	Control	OR	95%CI	p
		
	n = 561	%	n = 239	n = 322			
Level 1 – Demographic characteristics							
Household income *per capita* (MW)							0.236
> 1.0	50	8.9	20	30	1.00		
1.0–0.50	133	23.7	58	75	1.16	0.59–2.24	
0.5–0.25	222	39.6	85	137	0.93	0.49–1.74	
≤ 0.25	156	27.8	76	80	1.42	0.74–2.72	
Socio-economic class							0.033
B1 + B2 + C1	153	27.3	54	99	1.00		
C2	263	46.9	112	151	1.36	0.90–2.05	
D + E	145	25.8	73	72	1.85	1.16–2.95	
Education level							< 0.001
High school and university (C/I)	158	28.2	45	113	1.00		
Basic education (C)	195	34.8	79	116	1.71	1.09–2.67	
Basic education (I)/Illiterate	208	37.1	115	93	3.10	1.99–4.82	
Work							0.931
Yes	147	26.2	63	84	1.00		
No	290	51.7	125	165	1.01	0.67–1.50	
Has never worked	124	22.1	51	73	0.93	0.57–1.51	
Situation of current job							0.234
Formal contract	82	14.6	30	52	1.00		
Other	479	85.6	209	270	1.34	0.82–2.17	
Access to the Internet							< 0.001
Yes	437	77.9	168	269	1.00		
No	124	22.1	71	53	2.14	1.43–3.21	
Access to telephone							0.001
Yes	513	91.4	207	306	1.00		
No	48	8.6	32	16	2.95	1.58–5.52	
Age group (years)							0.365
≥ 30	133	23.7	56	77	1.00		
25–29	113	20.1	42	71	0.81	0.48–1.36	
20–24	159	28.3	76	83	1.25	0.79–2.00	
≤ 19	156	27.8	65	91	0.98	0.61–1.57	
Marital status							0.005
Married	51	9.1	14	37	1.00		
Common-law marriage	350	62.4	142	208	1.80	0.94–3.45	
Single/Divorced/Widow	160	28.5	83	77	2.84	1.43–5.67	
Race (self-reported)							0.200
White	97	17.3	36	61	1.00		
Non-white	467	82.7	203	261	1.31	0.84–2.06	

C: complete; I: incomplete

Table 2 shows the behavioral variables. Religion, age at first intercourse and pregnancy, number of pregnancies and sexual partners, and age at which started smoking, drinking, and using illicit drugs were significant. The following variables were also considered: the condition of the current partner regarding the use or not of illicit drugs, access to information, and situation of conflict.

**Table 2:** Bivariate analysis of behavioral factors for syphilis in women. Recife, State of Pernambuco, Brazil, 2014.

Variable	Total		Case	Control	OR	95%CI	p
		
	n = 561	%	n = 239	n = 322			
Level 2 – Behavioral characteristics							
Religion							0.044
No religion	208	37.1	92	116	1.00		
Catholic	177	31.6	85	92	1.16	0.77–1.74	
Evangelical	176	31.4	62	114	0.68	0.45–1.03	
Age in the 1st sexual intercourse (years)							0.002
≥ 18	114	20.3	34	80	1.00		
≤ 17	447	79.7	205	242	1.99	1.28–3.10	
Age in the 1st pregnancy (years)							< 0.001
≥ 18	302	53.8	108	194	1.00		
≤ 17	259	46.2	131	128	1.83	1.31–2.57	
Number of pregnancies (previous + current)							0.002
1	187	33.3	62	125	1.00		
2–3	256	45.6	115	141	1.64	1.11–2.43	
≥ 4	118	21.0	62	56	2.23	1.39–3.58	
Number of sex partnersa							< 0.001
1	434	77.4	161	273	1.00		
2	75	13.4	40	35	1.93	1.18–3.17	
≥ 3	52	9.3	38	14	4.60	2.42–8.75	
Condom use							0.338
Always	60	10.7	29	31	1.00		
Sometimes	186	33.2	84	102	0.88	0.49–1.57	
No	315	56.1	126	189	0.71	0.40–1.24	
Age at which started smoking (years)							< 0.001
Does not smoke	449	80.0	166	283	1.00		
≥ 18	19	3.4	9	10	1.53	0.61–3.85	
≤ 17	93	16.6	64	64	3.76	2.33–6.07	
Age at which started drinking (years)							0.144
Does not drink	453	80.7	187	266	1.00		
≥ 18	30	5.3	11	19	0.82	0.38–1.77	
≤ 17	78	13.9	41	37	1.57	0.97–2.55	
Age at which started using drugs (years)							< 0.001
Does not use	480	85.6	181	299	1.00		
≥ 18	19	3.4	10	9	1.83	0.73–4.60	
≤ 17	62	11.1	48	14	5.66	3.03–10.56	
Current partner uses drugs							< 0.001
No	454	80.9	176	278	1.00		
Yes	107	19.1	63	44	2.26	1.47–3.47	
Suffers discriminationa							0.731
No	435	77.5	187	248	1.00		
Yes	126	22.5	52	74	0.93	0.62–1.39	
Watches television							0.426
Everyday	483	86.1	209	274	1.00		
Sometimes a week	78	13.9	30	48	0.81	0.50–1.33	
Listens to the radio							0.499
Everyday	224	39.9	102	122	1.00		
Sometimes a week	217	38.7	87	130	0.24	0.54–1.16	
Never	120	21.4	50	70	0.49	0.54–1.33	
Access to informationb							0.140
Has improved	396	70.6	160	236	1.00		
Has remained the same	145	25.8	67	78	1.26	0.86–1.85	
Has worsened	20	3.6	12	8	2.21	0.88–5.53	
Situation of conflictb							0.070
No	434	77.4	176	258	1.00		
Yes	127	22.6	63	64	1.44	0.97–2.14	

^a^ In the last 12 months.

^b^ In the last 5 years.

Among the variables related to clinical, obstetric, and care history, we selected for the adjustment step in the multivariate analysis: number of appointments during prenatal care, previous occurrence of sexually transmitted infection, participation in group of pregnant women, record in family health unit, and HIV serology in the maternity hospital (Table 3).

**Table 3:** Bivariate analysis of factors related to clinical, obstetric, and health care history in prenatal care and maternity hospital for syphilis in women. Recife, State of Pernambuco, Brazil, 2014.

Variable	Total		Case	Control	OR	95%CI	p
		
	n = 561 %		n = 239	n = 322			
Level 3 – Clinical, obstetric, and health care history in prenatal care and maternity hospital							
History of previous abortion							0.001
No	406	72.4	155	251	1.00		
Yes	155	27.6	84	71	1.91	1.31–2.78	
History of previous STI							< 0.001
No	434	77.4	135	300	1.00		
Yes	127	22.6	105	22	10.68	6.46–17.66	
Gestational age when prenatal care began (trimester)							< 0.001
First	323	57.6	112	211	1.00		
Second	149	26.6	68	81	1.58	1.06-2.34	
Third	17	3.0	11	6	3.45	1.24-9.58	
No prenatal care	72	12.8	48	24	3.76	2.19-6.47	
Number of appointments in prenatal care							< 0.001
≥ 7	217	38.7	67	150	1.00		
6–4	178	31.7	66	112	1.31	0.86–2.00	
3–1	94	16.8	58	44	3.60	2.17–5.98	
No prenatal care	72	12.8	48	24	4.47	2.53–7.90	
Place of prenatal care							< 0.001
FHP	273	48.7	109	164	1.00		
Other	216	38.5	82	134	0.92	0.63–1.32	
No prenatal care	72	12.8	48	24	3.00	1.74–5.19	
Received prenatal card in the 1st appointment							< 0.001
Yes	454	80.9	178	276	1.00		
No	35	6.2	13	22	0.91	0.45–1.86	
No prenatal care	72	12.8	48	24	3.10	1.83–5.24	
Exams were requested in the 1st prenatal appointment							< 0.001
Yes	463	82.5	176	287	1.00		
No	26	4.6	15	11	2.22	0.99–4.95	
No prenatal care	72	12.8	48	24	3.26	1.93–5.51	
Participated in the group of pregnant women*							< 0.001
Yes	103	18.4	38	65	1.00		
No	386	68.8	153	233	1.12	0.71–1.76	
No prenatal care	72	12.8	48	24	3.42	1.81–6.44	
Record at the FHU							0.156
Yes	398	70.9	162	236	1.00		
No	163	29.1	77	86	1.30	0.90–1.88	
HIV serology at the maternity hospital							0.017
Negative	541	96.4	225	316	1.00		
Positive	20	3.6	14	6	3.27	1.24–8.65	

STI: sexually transmitted infection; FHP: family health program; FHU: family health unit; HIV: human immunodeficiency virus

* In the current pregnancy.

Table 4 shows the results of the multivariate analysis, including all variables analyzed. Previous history of sexually transmitted infection (OR = 9.7; 95%CI 5.4–17.2) and only one to three appointments during prenatal care (OR = 3.5; 95%CI 1.8–6.6) were the factors associated with a higher risk for syphilis. Women who had three or more sexual partners in the last year were three times more at risk for infection. A similar finding was found among those who started using drugs before the age of 18 (OR = 3.0; 95%CI 1.4–6.4). The lack of access to telephone was associated with syphilis (OR = 2.4; 95%CI 1.2–4.7). Among those with a lower education level, the presence of syphilis had OR of 2.02 (95%CI 1.1–3.4).

**Table 4:** Multivariate logistic regression of sociodemographic and behavioral factors, and clinical, obstetric, and health care history in prenatal care and maternity hospital for syphilis in women. Recife, State of Pernambuco, Brazil, 2014.

Variable	Unadjusted			Adjusted		
	
	OR	95%CI	p	OR	95%CI	p
Level 1^a^ – Demographic characteristics						
Education level			< 0.001			0.024
High school and university (C/I)	1.00			1.00		
Basic education (C)	1.71	1.09–2.67		1.27	0.73–2.19	
Basic education (I)/Illiterate	3.10	1.99–4.82		2.02	1.17–3.47	
Access to telephone			0.001			0.006
Yes	1.00			1.00		
No	2.95	1.58–5.52		2.45	1.28–4.70	
Level 2^b^ – Behavioral characteristics						
Religion			0.044			0.012
No religion	1.00			1.00		
Catholic	1.16	0.77–1.74		1.70	1.07–2.68	
Evangelical	0.68	0.45–1.03		0.86	0.53–1.39	
Number of pregnancies including current one			0.002			0.008
1	1.00			1.00		
2–3	1.64	1.11–2.43		1.77	1.12–2.79	
≥ 4	2.23	1.39–3.58		2.27	1.30–3.95	
Number of sex partners in the last 12 months			< 0.001			0.004
1	1.00			1.00		
2	1.93	1.18–3.17		1.66	0.93–2.97	
≥ 3	4.60	2.42–8.75		3.15	1.51–6.53	
Age at which started using drugs (years)			< 0.001			0.003
Does not use	1.00			1.00		
≥ 18	1.83	0.73–4.60		0.50	0.16–1.53	
≤ 17	5.66	3.03–10.56		3.04	1.45– 6.40	
Current partner uses drugs			<0.001			0.026
No	1.00			1.00		
Yes	2.26	1.47–3.47		1.78	1.07–2.98	
Level 3^c^ – Clinical, obstetric, and health care history in prenatal care and maternity hospital						
Number of appointments in the prenatal care			< 0.001			< 0.001
≥ 7	1.00			1.00		
6–4	1.31	0.86–2.00		1.48	0.88–2.49	
3–1	3.60	2.17–5.98		3.53	1.86–6.69	
No prenatal care	4.47	2.53–7.90		3.20	1.36–7.54	
History of previous STI			< 0.001			< 0.001
No	1.00			1.00		
Yes	10.68	6.46–17.66		9.70	5.46–17.24	

C: complete; I: incomplete; STI: sexually transmitted infection

^a^ Odds ratio adjusted for all variables of level 1 with p ≤ 0.20.

^b^ Odds ratio adjusted for all variables of level 1 and variables of level 2 with p ≤ 0.20.

^c^ Odds ratio adjusted for all variables of level 1 and 2 and variables of level 3 with p ≤ 0.20.

There was a higher risk of syphilis among multiparous women (OR = 2.2; 95%CI 1.3–3.9) and among those who reported that the current partner is a user of illicit drugs (OR = 1.7; 95%CI 1.0–2.9). Religion was also associated with the outcome; the category “no religion” was used as a reference and those affiliated with the Catholic religion presented the highest risk (OR = 1.70; 95%CI 1.0–2.6).

## DISCUSSION

In this study, we identified that poverty and its associated vulnerability – be it behavioral or the access and quality of prenatal care offered in health services – are significantly associated with syphilis in pregnant women. This result indicates that the control of the transmission remains an unsolved challenge, in line with what has been observed in other investigations^8,15,18^. Although syphilis presents simple diagnostic and treatment methods, it remains a health and social problem worldwide^17,21^.

This study has limitations that should be pointed out. As the data were collected with a face-to-face interview, there is a possibility of classification errors, mainly for behavioral variables, such as condom use, alcohol use, and number of sexual partners. On the other hand, there is no evidence of differential classification error between cases and controls. As neither interviewer nor respondent knew who was reactive and non-reactive to ELISA at the time of the interview, these classification errors are non-differential, tending to make the groups more similar and to underestimate the associations found.

Regarding the characteristics of health care in prenatal care and maternity hospital, a methodological issue must be discussed: the category of “no prenatal” was repeated in six variables analyzed, influencing the exclusion and power of the other ones in the adjustments. The variable of “number of prenatal appointments” was chosen because of its stability in different adjustments and because it can consolidate the set of actions established by the prenatal care program.

Estimates of social inequalities in health in Brazil support the hypothesis that gestational syphilis is associated with low socioeconomic status and inadequate prenatal care, contributing to the persistence of vertical transmission and exposing the fragility of the care not only in terms of access, but also in the opportunity for screening, diagnosis, and treatment of pregnant women and their partners^17,23^. It is known that unsafe sexual practices and lack of social support increase the risk of recurrent infections^15,17^.

The variables that showed the greatest strength of association for gestational syphilis in this study were previous history of sexually transmitted infection, followed by an inadequate number of prenatal appointments (one to three appointments). It is well known that syphilis is one of the sexually transmitted infections that cause greater damage to pregnant women and their unborn children, and it is associated with an increased risk of HIV infection^21^.

The low frequency of prenatal appointments, indicated as responsible for several diseases during pregnancy, presented a risk of 3.5 for syphilis. The category of “no prenatal care” showed a similar risk (OR = 3.2). This shows the close relationship between the adverse effects on pregnancy and the type of care provided in prenatal care, and it reveals that the continuity of care with detection procedures for the risk of syphilis is important for its effectiveness. Much has been discussed about the protective role of prenatal care, which can also minimize the effects of socioeconomic inequalities^1,9,14^. Our results indicate that women with low frequency of prenatal appointments are at higher risk for syphilis, which decreases as the number of appointments increases.

Although these findings are not unexpected, they show aspects that need to be discussed by all actors in prenatal care; the lack of or non-compliance with the minimum routine recommended by the Ministry of Health, including counseling, epidemiological surveillance, pharmacological and laboratory actions, and the screening of partners compromise the control of the transmission of syphilis^16^. The loss of these “opportunities” produces a less effective performance of the health team and services, which fail to ensure comprehensive care, with repercussions on the pregnant woman and the unborn child.

The number of sexual partners in the last year was associated with the outcome studied. Population studies in Brazil show that the earlier the onset of sexual life, the greater the number of partners, as well as the chances of health risk^22^. Among Brazilian women, the average number of sexual partners in the last 12 months decreases as education level increases, a condition considered as a process of self-protection^22^. It is important to emphasize that the actions proposed for the control of syphilis among women still have the challenge of articulating prevention and care regarding the promotion of sexual and reproductive autonomy.

We did not approach the sexual partners in this research. Some variables were investigated with the women. The use of illicit drugs showed an association with the occurrence of syphilis in women. Regardless of the limitations, the questions were related to the current partner. The results were important because they broadened the look to other behavioral characteristics and were not limited to the adequacy or inadequacy of the treatment received. Among women, the use of illicit drugs before the age of 18 was also associated with syphilis.

It is possible that the increased incidence of infection may be explained by situations of vulnerability, ranging from behavioral and reproductive characteristics to unsafe sexual practices^5,7,19^. The behavioral profile of partners deserves more specific studies that highlight situations that are still exclusionary, such as the use of other drugs, insufficient adherence to accompany women to prenatal appointments, and inadequate reception in health services^4^.

Lack of access to telephone and low education level showed an association with the presence of syphilis, a condition that has been explored in several studies^13,15,18,23^. Access to telephone was analyzed as a sociodemographic variable because it represents individual purchasing power. Additionally, in our sample, there was a higher occurrence of syphilis in women from the most disadvantaged social strata and with more vulnerable lifestyles. However, the adoption of safe sexual behaviors is admittedly complex, not depending solely on education level, income, access to information, and materials such as condoms, but also on the meanings attributed to sexuality and health self-care^13,18^.

The high number of pregnancies was associated with the occurrence of syphilis. In Brazil, since the 1970s, there has been a reduction in the fertility rate among women. However, the impact varies because of sociodemographic factors, lower education level, absence of partners, drug use, early sexual life, and no prenatal care^1,13,25^.

It is also worth noting that the proportion of women who give birth and who are not submitted to the recommended treatment for the control and prevention of vertical transmission of syphilis is high; consequently, infection persists from one pregnancy to another with a greater chance of outcomes adverse to the unborn child, such as abortion, prematurity, and death^17^. Appropriate prenatal care could not only reduce incidents of syphilis during pregnancy with counseling and encouragement of condom use, but also with the appropriate treatment of prevalent cases in future pregnancies^11^.

Affiliation with the Catholic religion was associated with syphilis. Although there are studies that indicate that religiosity tends to delay the onset of sexual life, the interrelationships between the variable and sexual behavior are still little explored in the scientific literature and further research in this direction is necessary in Brazil^20^.

Although not detected in this sample, other studies have found an association between the lack of use of condoms and the growth of sexually transmitted infections^13,22^. The high frequency of stable relationships in this population may have contributed with the unprotected sex, probably because of greater affection and confidence in the partner, relativizing measures of prevention for infections and pregnancy. In addition, condoms are still seen as a symbol of infidelity or mistrust and should be used only in relationships with “unknown” partners^5,22^.

Campos et al.^4^ consider, regarding these difficulties on the use of condoms, that health services should adopt a differentiated approach, also during prenatal care, that favors the reception and identification, together with the women, of negotiation strategies with the partner, since reinfection can perpetuate syphilis.

Age group was not associated with syphilis, although national and regional research studies^3,9,15,23,24^ carried out in the last 10 years have found a higher occurrence of infection among women with a mean age of 23 years and adolescents. Although the findings cannot be extrapolated to other locations, considering the characteristics of the sample, we can highlight the dissemination of the disease and the need to expand education and prevention actions to reach women of all age groups, including those with greater vulnerability. In the last decades, there have been significant sociodemographic changes in Brazil, which implies new behaviors in relation to the regulation of sexual life^20^. However, this variable should continue to be investigated in studies of risk factors that include a greater number of participants.

The associations found indicate the need for the reorganization of actions, prioritizing women with the risk characteristics identified. Strategies aimed at these populations can be considered the starting point of the process and they are fundamental for reducing the vertical transmission of syphilis.
